# Adaptation of a Smartphone-Based Mobile Health Program to Support Person-Centered Treatment of Tuberculosis in Kilimanjaro, Tanzania: Preimplementation Qualitative Needs Assessment

**DOI:** 10.2196/92051

**Published:** 2026-06-01

**Authors:** Kennedy Ngowi, Liza Khutsishvili, Carolin Fabian, Margaretha Sariko, Krisanta Wilhelm, Stellah Mpagama, Karen Ingersoll, Scott Heysell, Jacqueline Hodges

**Affiliations:** 1 Department of Clinical Trials, Kilimanjaro Clinical Research Institute, Moshi, Kilimanjaro, United Republic of Tanzania; 2 Department of Medicine, University of Virginia, Charlottesville, VA, United States; 3 Infectious Diseases Unit, Kibong’oto Infectious Diseases Hospital, Kilimanjaro, Siha, United Republic of Tanzania; 4 Department of Medicine, Duke University, 315 Trent Drive, Durham, NC, 22710, United States, 1 919 681 1309

**Keywords:** mobile health, digital health, smartphone, tuberculosis, human immunodeficiency virus, implementation science, resource-constrained settings, mobile phone

## Abstract

**Background:**

Despite increasing smartphone penetration worldwide, personalized mHealth (mobile health) care interventions remain largely untapped for the support of people with tuberculosis. An evidence-based multifeature smartphone platform for HIV care tailored and widely implemented in the United States may enhance treatment quality and completion in the Kilimanjaro context.

**Objective:**

We aimed to evaluate contextual determinants of mHealth implementation in the Kilimanjaro region to ensure feasibility, acceptability, and effective adaptation of the platform for tuberculosis care within Kilimanjaro.

**Methods:**

We conducted semistructured in-depth interviews at Kilimanjaro Christian Medical Centre and Kibong’oto Infectious Diseases Hospital with people with tuberculosis (aged 18+ years with drug-susceptible/-resistant tuberculosis, with or without HIV, and >1 mo on treatment) and providers and staff (eg, clinicians, community health workers, or laboratory staff). Interview guides were designed using Bury’s Framework for Chronic Illness and the Consolidated Framework for Implementation Research, along with an overview of an existing smartphone-based program called PositiveLinks. Interviews were analyzed using thematic analysis, and determinants were mapped to behavior change frameworks to develop a mechanistic understanding of *P* adaptation for the context.

**Results:**

We conducted 14 interviews with people with tuberculosis and 11 provider and staff interviews. Several unmet tuberculosis treatment needs emerged, along with suggestions for platform adaptation and implementation strategies. Findings suggest high personal smartphone access among providers and staff (11/11, 100%), less so for people with tuberculosis interviewed (5/14, 36%). High provider digital literacy and capability and usage were noted, with smartphone apps routinely used for tuberculosis care delivery independent of electronic health systems. People with tuberculosis primarily used mobile phones for communication (calls) with clinic providers and staff for care coordination (eg, reminders). Internet access and stability remain major barriers in rural clinics, along with the personal cost of data bundles for both stakeholder groups. Key assets identified within the inner setting of Kilimanjaro Christian Medical Centre and Kibong’oto Infectious Diseases Hospital include existing provider and staff commitment to treatment support outside of clinic visits, and a robust infrastructure of community outreach for support of adherence and retention for people with tuberculosis.

**Conclusions:**

Findings suggest a role for broader digital wraparound support beyond adherence monitoring for tuberculosis care in the context. Real-world considerations for the context suggest implementation of provider-facing smartphone interventions was perceived as highly feasible and acceptable, with appropriate consideration of personal cost associated with usage among stakeholders. Patient-facing or bidirectional tools would require modifications to existing mHealth implementation strategies, including more comprehensive assessment of digital literacy and related training, as well as provision of subsidized devices and data bundles.

## Introduction

Tuberculosis remains the leading infectious cause of mortality worldwide [1]. Tanzania remains among the 20 countries with the highest estimated incidence of tuberculosis and comorbid HIV and tuberculosis burden [2]. Treatment for tuberculosis is demanding, with strict medication adherence necessary to ensure treatment response and prevent relapse, often associated with significant side effects [3]. Tuberculosis remains a highly stigmatized illness, with additional barriers and poorer treatment outcomes identified for those with HIV and tuberculosis coinfection [4] and multidrug-resistant tuberculosis, including in the Kilimanjaro region [5].

mHealth (mobile health) interventions have been increasingly studied to enhance patient-centered care, with various forms of smartphone apps piloted for HIV and tuberculosis care across a wide variety of settings, including in Sub-Saharan Africa, where penetration of feature phones as well as smartphones has increased substantially over time [6]. To date, person-centered mHealth (mobile phone) interventions developed for tuberculosis management primarily focus on enhancing patient self-management, including medication adherence, monitoring, and reminders. Variable uptake, usage, and clinical efficacy have been demonstrated when interventions are scaled among larger populations [7-9], including in Africa and other contexts [10,11]. Demonstrated impact on tuberculosis care in real-world settings is limited [9,12-14], and few interventions to date encompass multiple features for complex patient self-management, care coordination, and enhanced support [15].

**Figure 1.**
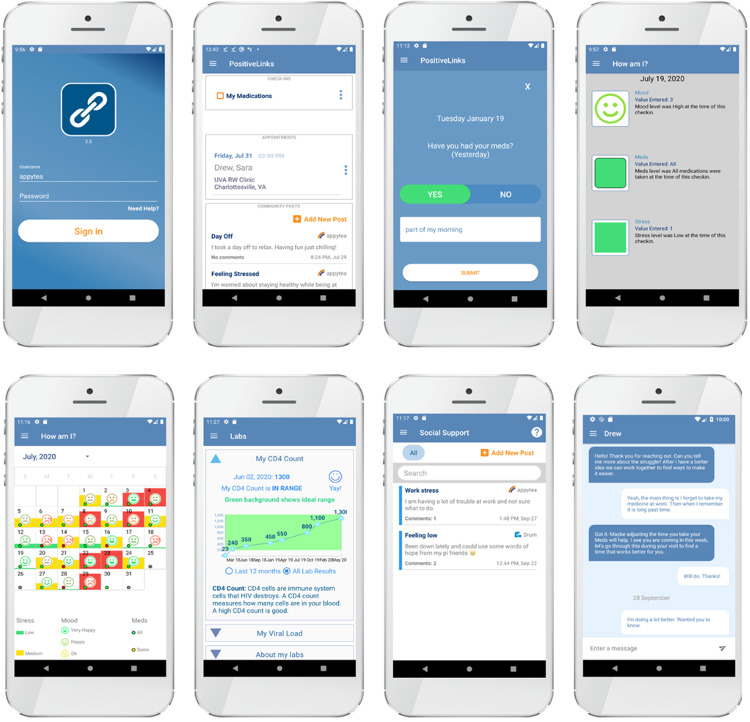
Screenshots of the most updated version of the PositiveLinks platform, demonstrating various features considered for adaptation in the context. Eight screenshots demonstrating each feature of the existing PositiveLinks platform (a secure login page, a homepage with a dashboard overview, a daily “check-in” prompt for medication administration, a dashboard to review check-ins for mood, stress, and medications along with a calendar view of responses for self-monitoring, a laboratory result dashboard for reviewing result trends over time with a figure, peer message board groups, and the patient-provider in-app messaging feature with chat bubbles). CD4: cluster of differentiation 4; UVA RW: University of Virginia Ryan White.

Smartphone-based digital platforms have not been well studied beyond assessments of feasibility and acceptability to support care of either HIV or tuberculosis within Africa [6,15,16]. A multifeature smartphone platform developed by our research group, PositiveLinks (Figure 1), has a strong evidence base for improving retention in care and care engagement for people with HIV in the United States [17-20]. PositiveLinks was designed through the application of two theoretical frameworks. Social action theory (SAT) [21] incorporates contextual influences on interacting self-regulation and social relationships to influence self-management and change behaviors. The information-motivation-behavior (IMB) skills model [22,23] emphasizes self-efficacy for behavior change, whereby individuals must understand information, build motivation for action, and deploy behavioral skills to change health behaviors. The platform incorporates features designed based on SAT and IMB theory, beyond monitoring and encouraging medication adherence, to enhance self-management, social or peer support, and care coordination [17,19]. The platform was further adapted for a pilot cohort with tuberculosis and HIV in the high-burden region of Irkutsk, Siberia, with high rates of platform engagement and improvement in clinical outcomes compared to historical controls [9,24]. Drivers of intervention uptake and implementation, however, were not well characterized for this cohort, and this context is highly distinct from those encountered within Tanzania.

Despite increasing personal smartphone access worldwide, personalized mHealth care interventions remain largely untapped for the support of people with tuberculosis. An evidence-based multifeature smartphone platform for HIV care tailored and widely implemented in the United States may enhance treatment quality and completion in the Kilimanjaro context. Evaluation of contextual influences on mHealth implementation is needed to ensure feasibility, acceptability, and effective adaptation for tuberculosis care within Kilimanjaro.

In this paper, we describe a contextual assessment conducted among stakeholders in the Kilimanjaro region of Tanzania to understand potential determinants of implementation of an mHealth, smartphone-based strategy to deliver multidimensional, person-centered care of people with tuberculosis within the context. We applied validated implementation science methodology to evaluate the feasibility and acceptability of adapting our evidence-based mHealth platform and program for the context, as well as to identify actionable targets for platform and program modification to appropriately tailor them for the Kilimanjaro context.

## Methods

### Study Setting

This qualitative study was conducted among stakeholders at 2 clinical sites in Northern Tanzania, the Kilimanjaro Christian Medical Centre (KCMC) in Moshi and the Kibong’oto Infectious Disease Hospital (KIDH) in Siha. At both facilities, patients may be admitted for the introduction of intensive phase treatment and then discharged once healthy enough to complete continuation phase treatment at district or regional health facilities, either with facility-directed or home-based directly observed therapy (DOT) with a treatment supporter [25,26]. Most patients do not require admission, and when admitted, the duration is usually days to weeks until clinical stabilization.

### Recruitment

Patients were recruited from KIDH and KCMC using convenience sampling, with purposive sampling of at least 5 individuals with HIV and tuberculosis coinfection. For providers, purposive sampling was used to capture diverse roles, employment duration, and site, with recruitment continued in parallel with the coding process until adequate thematic saturation was reached [27]. Eligible patients were (1) 18 years or older, (2) diagnosed with drug-susceptible or multidrug-resistant tuberculosis, and (3) undergoing treatment for tuberculosis (for at least one month) at the time of enrollment, or completed treatment within the past 5 years. Eligible patients identified by site clinic staff were approached by study staff for participation, provided study background, and enrolled in this study, either by telephone outreach (up to 3 attempts) or in-person during routine clinic visits. Providers, clinic, and hospital staff (including clinicians, support staff, laboratory staff, and other patient-facing roles) were identified through referrals from collaborating site clinicians and leadership and were approached either in-person or by phone.

### Data Collection

Semistructured in-depth interview guides were developed using Bury’s framework for chronic illness [28,29], which examines the impact of chronic illness on daily life, relationships, expectations, and identity. Additional probes were adapted from prior formative studies of the PositiveLinks platform and related prior adaptations (eg, smartphone and internet access, familiarity, or usage; perspectives on existing features and additional possible features) [9,17,30]. Provider interview guides applied the Consolidated Framework for Implementation Research (CFIR) [31,32] to probe for context-specific implementation determinants [33]. Participants completed a brief baseline demographic survey at the start of the interview. Interviews were conducted in Kiswahili. Translation was conducted by 3 trained study team members (KN, KW, and MS) fluent in the language as well as English. All translations were subsequently cross-checked for accuracy and consistency by a senior member of the research team (KN). During interviews, participants were given a descriptive verbal overview of the PositiveLinks platform and existing features based on a standardized script, with corresponding interview guide probes related to perceptions of core features (self-monitoring including medications, laboratory results, stress and mood check-ins, appointment reminders, in-app communication, and community chat board). Interviews were conducted by a study team member in person at KCRI and Kibong’oto Hospital. Demographic information was collected at the beginning of each interview.

### Data Analysis

Interviews were audio-recorded, transcribed, and translated by KCRI study team members and were uploaded to qualitative analysis software NVivo (version 14.23.2; Lumivero). A preliminary codebook was developed by study team members deductively from the applied frameworks and iteratively refined with emergent inductive codes. All transcripts were coded by 2 study team members, including at least 1 senior researcher (JH and KN). Coding discrepancies were resolved through consensus among the coding team until intercoder agreement (κ score of 61%, indicating substantial agreement) [34,35] was reached consistently (double-coding of 11/25, 44%, of the transcripts) for all coding pairs. This threshold was selected based on the breadth of the interview guide topics and the exploratory nature of the current study [34]. The κ score was calculated in NVivo using coding comparisons. The coding team met regularly to review codes and related analytic memos throughout the coding process, noting observations, preliminary patterns, and theories in the data, as well as discussing potential implications of reflexivity across the diverse coding team with distinct backgrounds and perspectives [36]. These reflections were applied with further iteration of the codebook until thematic saturation was achieved. Thematic analysis was performed by 2 formally trained qualitative researchers on the current study’s team (KN and JH) [37]. The current study’s team prioritized themes and subthemes that were commonly identified and conceptually relevant. Illustrative supportive quotes were selected based on team consensus. We then mapped themes to the IMB skills model and SAT to develop a mechanistic understanding of PositiveLinks adaptation for the context, including “platform-modifiable” contextual determinants identified as either a facilitator or barrier to implementation, or impacting implementation outcomes positively or negatively (eg, related platform feature usability or acceptability).

### Statistical Analysis

Descriptive statistics were used to summarize baseline demographic data from surveys completed by participants at the start of the interview. Baseline variables of interest were summarized as sample means or proportions for each respondent type (people with tuberculosis or providers and staff members). Statistical analysis was performed using Microsoft Excel (Microsoft Corp) and R (version 4.1.2; R Foundation).

### Ethical Considerations

All study procedures were conducted according to a protocol approved by the National Institute of Medical Research, Tanzania (NIMR/HQ/R.8a/Vol. IX/4328) and the University of Virginia Institutional Review Board (IRB HSR230405). Written informed consent was obtained for all study participants using procedures outlined in the institutional review board–approved study protocol. Data collection, storage, and management followed all outlined procedures (eg, deidentification of baseline survey data and use of an assigned study ID with a separate link log). No monetary compensation was provided to participants for study activities described in this paper.

## Results

### Overview

**Figure 2.**
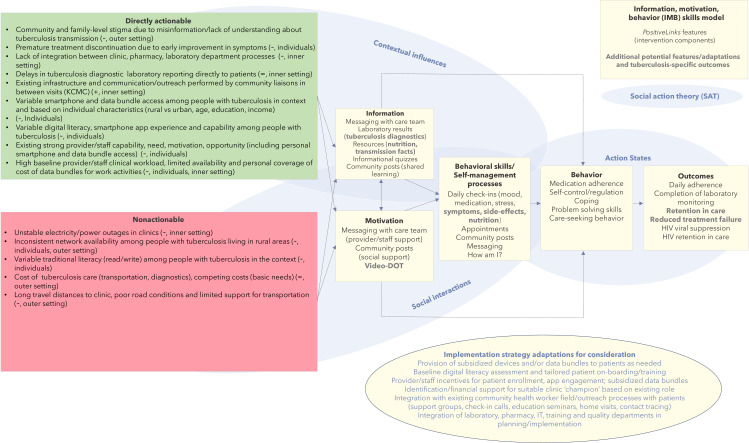
Summary of emergent themes from in-depth interviews with providers and patients mapped to the IMB skills model and SAT. Contextual determinants are described as either actionable, based on preimplementation tailoring of the care platform/implementation strategy for tuberculosis care in the context, or nonactionable (determinants that cannot be realistically adjusted with modifications to the platform or implementation strategy ahead of implementation). Key implementation determinants are further categorized as a barrier (−), facilitator (+), or neutral (=; suggestion/feedback provided without valence) in relation to PositiveLinks implementation based on interview analysis, and by respective CFIR domain as appropriate. The resulting feature modifications based on qualitative feedback, including highlight determinants as well as feature-specific feedback on probing (eg, CFIR domain of innovation) for consideration and ranking, are noted among the PositiveLinks features already available and mapped to both IMB and SAT. CFIR: Consolidated Framework for Implementation Research; DOT: directly observed therapy; IMB: information-motivation-behavior; KCMC: Kilimanjaro Christian Medical Centre; SAT: social action theory.

A total of 14 people with tuberculosis were enrolled from KIDH (n=8, 57%) and KCMC (n=6, 43%; Table 1). A total of 11 providers and staff members were enrolled from KIDH (n=5, 50%) and KCMC (n=6, 55%). Emergent themes are summarized for people with tuberculosis and provider and staff interviews in Figure 2, and findings are described further below.

**Table 1.** Baseline demographic data for people with tuberculosis (n=14) and providers/staff (n=11) participating in in-depth interviews. Duration describes years since the diagnosis of tuberculosis was documented (patient) and employment at the site (providers/staff).

Baseline demographic characteristics	Values
People with tuberculosis (n=14)	
Site, n (%)	
Kibong’oto Infectious Diseases Hospital	8 (57)
Kilimanjaro Christian Medical Centre	6 (43)
Sex, n (%)	
Male	9 (64)
Female	5 (36)
Age (years), mean (SD)	43 (11)
Education level, n (%)	
Primary	8 (57)
Secondary	2 (14)
University	0 (0)
Graduate	1 (7)
Unavailable	0 (0)
Duration (since diagnosis date, years), mean (SD)	2.9 (5.0)
Residence (district), n (%)	
Moshi Urban	6 (43)
Moshi Rural	2 (14)
Hai	2 (14)
Siha	1 (7)
Rombo	0 (0)
Mwanga	0 (0)
Same	0 (0)
Other region	2 (14)
Occupation, n (%)	
Farming	4 (29)
Transportation	3 (21)
Business	2 (14)
Hospitality	2 (14)
Mining	1 (7)
Other/unemployed	2 (14)
Infection type, n (%)	
Tuberculosis mono-infection	6 (43)
HIV/tuberculosis	8 (57)
Providers/staff (n=11)	
Site	
Kibong’oto Infectious Diseases Hospital	5 (50)
Kilimanjaro Christian Medical Centre	6 (55)
Sex, n (%)	
Male	6 (55)
Female	4 (36)
Age, mean (SD)	42 (13)
Education level, n (%)	
Primary	0 (0)
Secondary	0 (0)
University	5 (40)
Graduate	2 (30)
Unavailable	4 (36)
Duration (employment, years), mean (SD)	11 (7.4)
Residence (district), n (%)	
Moshi Urban	3 (27)
Moshi Rural	0 (0)
Hai	4 (36)
Siha	0 (0)
Rombo	0 (0)
Mwanga	0 (0)
Same	0 (0)
Other region	0 (0)
Occupation, n (%)	
Laboratory scientist	4 (36)
Nurse	2 (18)
Physician	1 (9)
Quality officer	1 (9)
Community liaison	1 (9)
Information technology	1 (9)
Hospital attendant	1 (9)

### Daily Life and Experience With Illness: People With Tuberculosis

People with tuberculosis described several challenges shaping their illness and treatment experiences, and related impacts on daily life (Table 2).

**Table 2.** Daily life and experience with illness: patient interview themes. Themes are listed in descending order with subthemes and supportive quotations, based on the number of participant interviews coded with the theme (I=interviewer, R=respondent).

Theme: daily life and experiences with illness	Illustrative quotes
Challenges	
Lack or loss of employment or financial challenges	“Things which changed were for example going in the farm to search for the livestock grass, it was not that far, it was like three or four kilometers. For this time I’m not capable of carrying that load as I used to…. your economic activities have being affected I mean that; maybe you were able to carry the livestock grass previously, you were able to feed your chicken well and also you were able to look for a job and earn money but for this time since you got this challenge in one way or other (your job) has been affected” (KIDH^a^).
Disclosure of diagnosis	“I didn’t tell all of them; this one right here (daughter) was the one I consulted so they could help me in this situation. She was very sad, but later on... she had to turn and accept it only” (KCMC^b^).
Physical isolation	“Yes, they were sleeping in their room and I was sleeping in my room... it was very scary because they would have died” (KIDH).
Quality of life, mental health	“You know, every day you go to this doctor, they just give you medicine, but when you take those medicines, you feel like they are not working. It’s like a waste of time, to the point of losing hope. I reached a point where I lost hope” (KIDH).
Stigma	“I don’t show anyone my medicine... If these people, like my friends, came to visit and saw that box with that mark, they wouldn’t enter at all” (KIDH).
Community, societal perceptions	I: “And how did they think about TB”? R: “They think it is a very bad disease especially when it comes to separation or lacking closeness to stay with them so it was a challenge.” R: “Well, there were elders who had this disease in our street and it was very scary because they died” (KIDH).
Illness course	
Medication adherence	“I bring the containers to the hospital, and they see that the medications have been taken. They are also calling by phone and saying, ’it’s time to take medications,‘ ’show us that you have taken them,’...If that is done, it helps to ensure that patients are taking their medications” (KIDH).
Patient understanding of illness, treatment	R: “So, you didn’t use the medicine for a time? I: I didn’t use for three months .I: Why? R:...what I saw was my situation has become good so I didn’t care again about going to take medication, and continue with my activities” (KCMC).
Experience with symptoms	“I was sick and while sleeping I was coughing, excessive sweating, then the coughing increased and I became weak until my body weight reduced and I had to take responsibility to go to the hospital, when I reached at the hospital, they took tests and discovered that I had TB, and started the treatments” (KIDH).
Treatment duration, response	“Currently it is different from previously, I feel good, I’m doing better... Now, I can work and the body does not weaken” (KCMC).
Tolerance to medication, side effects	“Aah, I just followed the way they were directing me…. I was told that these (medications) will bring me minor effects like rashes, itching and really when I used those medications, I experienced those conditions and they last for two to three weeks then they were over” (KCMC).
Living environment	“I live at my house in the village, not in town. We don’t have electricity, and transportation is quite limited. A road was built here, but it’s mostly unused because it’s rough. I live with my children and our goats, who are like family to us” (KCMC).
Frequency, manner of follow-up	“When you are done, maybe it is written on your card that you have to come on a certain day whereby those medications will be finished for example, day 1, day 2 up to day 7 when you are done with day 7, you return to the clinic so as you can be given another medication” (KCMC).
Tuberculosis transmission	“Since it was already in the family, we carried it as a family. Personally, I was protecting them well, when I discovered that I had TB, at my house there were no children, all of them were at school. When they came back, it’s like I was already starting taking medications for about a week so, when I asked a doctor that ’How can I protect my children since I have infections and I’m with the children?’ he told me first of all because I’m not coughing, it is not easy to affect them since the children are grownup, the other one is 13 years old and the other is older than him so, its not easy also because the child you should avoid is under 5 years so I was not going where there was a little baby” (KIDH).

a KIDH: Kibong’oto Infectious Disease Hospital.

b KCMC: Kilimanjaro Christian Medical Centre.

People with tuberculosis described significant losses of income and productivity following diagnosis, particularly for physically demanding jobs, resulting in reduced ability to support their households. Stigma was reported to influence many aspects of patients’ experience of illness, maintained by community members, leading to selective diagnosis disclosure, social isolation, lack or loss of support, and decreased quality of life and mental health. Protective behaviors such as physical separation within households increased feelings of isolation. Some individuals noted that after their diagnosis was disclosed, partners ended relationships and friends distanced themselves. Living environments were also described, with rural residents facing long travel distances and a lack of transportation. Housing (eg, overcrowded or poorly ventilated) and employment (eg, mining or farming) exposed some patients to harsh conditions and physical strain counter-productive to recovery.

People with tuberculosis described the frequency of follow-up visits (facility-based or home-based DOT) and facilitation by family members to coordinate follow-up visits and handle logistics. Patients cited various reasons for continuing medications, such as religious beliefs, responsibility to children, and witnessing the decline of peers who have gone untreated (poor health and unemployment). Facilitators for ongoing adherence included upfront counseling from the clinical team about side effects and the need to continue medications despite the absence of symptoms.

*Yes…the first time I started using the medicine, it made me feel very weak in my body… Surely, I was about to give up because it made me feel so weak. But one of my friends would tell me, “You will get used to it,” and when I gained experience with using it, I saw it was just a normal thing and continued to use it.*

[PWTB, KIDH]

### Determinants of Tuberculosis Care: People With Tuberculosis

The current factors describing how patients engage with care, including their experience with the quality of their treatment and their medication habits, are summarized in Table 3.

**Table 3.** Determinants of tuberculosis care, experiences with current care delivery, and medical needs. Themes are listed in descending order with subthemes and supportive quotations, based on the number of participant interviews coded with the theme (I=interviewer, R=respondent).

Theme	Illustrative quotes
Determinants of tuberculosis care	
Treatment supporters	I: ”And do you think being together with your fellow has helped you in taking medication, because we are saying that if you can create a group or be together is helping in reminding yourselves?” R: “Actually, 100% is me and her…We have finished (me and her). She has helped me very much compared to my wife because she was reminding on the date of returning (to the hospital), on taking medications and so on. Also, in comforting each other like how do you feel? so, we were cooperating in matters like those” (KIDH^a^).
Patient-provider relationship	“The answer that is short is that the doctor has told me ‘if you come we will test you,’ what sample will he take and what he will know... I was asking them to give me medication even for 2 months and they said you haven’t started yesterday, you have not done tests so go. If you come on 8th, we will test you for these medications that you have taken. If you have gone well, then we will give you these medications of 3 months” (KCMC^b^).
Access to communication with providers	“I communicate with people. Just like doctors, I tell them there is a changing condition, I must call him via a phone... Yes, he will tell me maybe do this or come quickly. It has a big contribution” (KIDH).
Level of motivation	“What motivated me to gain strength and take the medicine. Because I saw my friends had no jobs anymore, and I decided to make an effort to take the medicine to recover quickly” (KIDH).
Reminders (medications, appointments)	“They can call a patient by phone to ask how they are doing. If they notice that you missed a clinic appointment, they will call you to ask why you did not come. That’s how they follow up on patients” (KIDH).
Cost of care, incentives	“Yes, everything is really fine because something that was not making me give up, when I arrive there (at Kibong’oto) you are given your transport fee, you get the food, tea, soda.a person may fail to give up because when I come (at the hospital) and gets the transport fee that makes me to have strength to go (to the hospital)” (KIDH).
Personal access to test results	“When I go to the clinic, I come for a checkup. I must provide sputum. After giving the sputum, I submit it, then I measure my height, take my temperature, and afterward, I provide a blood sample…They check to see whether there are any changes in how I am taking my medications or not. I left my sputum there. I didn’t get the results from that sputum, but I finished the pills in March. I wasn’t shown the X-ray. Most of the time, at Kibong’oto, we were not given the results of the blood test; we only received medications” (KIDH).
Transportation access, distance to clinic	“It’s really far; it takes thirty shillings to get here... At the health center, my mom had to collect money from people (to seek support from family friends, neighbors etc) , some people from the church contributed, and I got support. That’s when I was brought here for the first time, especially for treatment, let’s say the second time” (KIDH).
Experiences with current care delivery and medical needs	
HIV tuberculosis integration	I: “And what if these services could be available together for example, when you enter... you get both services for TB and HIV?” R: “That could be good because you won’t go back, when you go there you take your stuffs together and leave...Yes, like now am taking [medication] for HIV then after that I go back to the first floor to take medications for TB but if they put them there it is not bad, they will be helping us” (KCMC).
Provider competency	“Surely, he (doctor) told me that you are already healed, then explained to him the way I am, so he told me that I will take the number from the doctor because I explained to him that there is a certain work I did and coughed blood so he told me there was a scar in the chest that was not yet healed and he told me to stop that work and after I stopped that work I have never coughed blood again but I’m still coughing much especially in the morning” (KIDH).
Timeliness, access to appointments	“When I reach there at the hospital, first of all I enter to the doctor at the reception, I provide the information of my card, they sign me in to the system, they give me the receipt then I go to the doctor, after going to the doctor he checks me and ask me some questions, I answer then he writes my information then he writes medications for me” (KIDH).

a KIDH: Kibong’oto Infectious Disease Hospital.

b KCMC: Kilimanjaro Christian Medical Centre.

The relationship between people with tuberculosis and providers plays a key role, with variable experiences reported around counseling on treatment needs, such as laboratory tests and complex medication routines. Most participants shared positive experiences with their providers, especially appreciating the ability to communicate directly with their doctors during times when symptoms worsened or their condition changed. Patients’ personal level of motivation/activation was described by several as an important determinant. On the other hand, perceived health improvements or treatment fatigue often caused patients to stop treatment early.

Daily medication adherence was supported by various reminders and personalized routines, such as aligning medication times with meals, setting phone alarms, using visual aids, writing detailed notes in treatment cards, exercise books, or notebooks, and seeking support from family members. Various clinic support strategies (SMS text reminders from the clinic or phone check-ins) and digital adherence technologies (alarm devices provided by the clinic, personal device alarms, or digital pill boxes) were described as initially helpful to improve accountability, with some noting they needed the reminder less as their treatment course went on.

While many aspects of tuberculosis treatment are subsidized by the government (eg, medications or physician services) [32], the cost of care remains a significant barrier (eg, transportation or follow-up care). Financial support services (eg, transportation vouchers, food, or stipends) facilitate visit attendance and were described to encourage continued engagement in care. Access to transportation and distance to clinics (ie, geographic remoteness) were critical determinants—people with tuberculosis reported selling assets or relying on social networks for related expenses, including relatives, neighbors, and religious communities:

*It’s really far; it takes thirty shillings to get here… At the health center, my mom had to collect money from people.*

[Patient, KIDH]

People with tuberculosis described various barriers related to personal access to test results, including delays from collection to release to the patient, lack of detail or direct access to results (eg, quantitative viral load counts, chest X-ray interpretations, etc). Several participants noted an inability to review results with their provider during medical visits due to inadequate internet connectivity. Patients who must physically return for laboratory results face unpredictable, long wait times once they arrive. Some participants reported that follow-up care done at local facilities makes receiving laboratory results easier, but facilities lack other complex care capabilities valued at KCMC and KIDH.

### Experiences With Current Care Delivery and Medical Needs: People With Tuberculosis

Key additional themes emerged relating to experiences with current care delivery and medical needs (Table 3). People with tuberculosis highlighted several positive aspects of care delivery, including flexibility (eg, family members can collect medications), clear communication, and positive rapport with their care team. Patients noted broader challenges encountered in the tuberculosis care continuum (delays in initial disease recognition, testing, and treatment initiation). Tuberculosis diagnoses often came after prolonged, misinterpreted symptoms and multiple clinical encounters.

Patients described variable access to integrated tuberculosis and HIV outpatient care—those accessing it (eg, at KCMC) expressed satisfaction with the coordination and efficiency of integrated care, appreciating access to a single provider managing both conditions, whereas integration was less commonly described at smaller, local facilities. Patients’ detailed experiences with provider competency, approach, and level of commitment generally describe care team members (physicians, nurses, or staff) as competent, trustworthy, and attentive. Patients reported positive experiences with counseling and education, noting that educational materials and seminars provided by the clinics were helpful. In one instance, however, inadequate anticipatory guidance led to premature self-discontinuation of treatment with resolution of symptoms.

### Smartphone Ownership, Experience, and Familiarity: People With Tuberculosis

Personal smartphone ownership was self-reported by 5 of 14 people with tuberculosis, 36% (5/14), with 3 of 8 patients with comorbid HIV reporting ownership (3/8, 38%). The remaining people with tuberculosis (n=9) instead reported owning feature phones (eg, “button phones” or “flip phones”). Most participants without personal ownership reported using a smartphone app at some point on another person’s phone (8/9, 89%). Nonowners described a perception of a typical smartphone owner as young-to-middle aged, employed, and well-educated. Some people with tuberculosis, without their own smartphones, noted that younger family members owned smartphones for school or work, and described borrowing a smartphone from a peer to use as needed.

People with tuberculosis noted that consistent internet connectivity was a challenge, particularly for rural residents, describing access primarily through increasingly expensive data “bundles.” Patients described mobile phone usage, primarily for communication with peers and family members (calling, texting, WhatsApp [Meta], or Facebook [Meta]), while others noted minimizing data usage due to cost (eg, urgent calls or checking the time). Specific usage for different aspects of tuberculosis care was described (communication with providers, setting phone alarms, and internet searches for disease-related information).

### Perceptions of Proposed mHealth Intervention and Features: Patients With Tuberculosis

Positive perceptions of acceptability of the proposed mHealth program and platform features were coded in 13 of 14 interviews (Table 4).

**Table 4.** Patient perceptions of proposed mHealth^a^ intervention features: themes and supportive quotations. Themes are listed in descending order by number of participant interviews coded with the theme, along with supportive quotations (I=interviewer, R=respondent).

Perceptions of proposed mHealth intervention for tuberculosis care	Illustrative quotes
Patient-provider communication	"For example, if I would like something, maybe to take a photo and send it to the doctor then the doctor could advise me... if there would be this system, it could help…You may have a sudden problem and not have the means to go... so you send the information and the doctor can advise you” (KIDH^b^).
Peer messaging board	"It will help because someone may get urgent information and share it in the group...If I ask a question and the doctor doesn’t reply, someone else might say ‘I had that, try this.’" (KIDH).
Reminders (appointment, medication)	"In my side I can say maybe the issue of reminder that he is required to return a certain day if possible in the forefront of telephone should show but also if possible, be able to notify him that there is clinic two days before, also able to remind him that he is required to use drugs daily” (KCMC^c^).
Self-monitoring check-ins	R: “Yes, because it can bring problems to a person.” I: “It might give a person pressure!” R: “Yes, so there must be an alternative thing in which even if a person is not in the mood will show him in a way that does not bring any challenge to him” (KIDH).
Educational resources	“It’s just like the way I told you when I got reactions and use my telephone to search in google, and me with my doctor we have planned a certain medications and if there is possibility for him to install them to me in the phone, that I have given you this and this and it work like this and this! So I will be able to look on it and when I encounter any challenge I can consult him” (KCMC).
Laboratory results dashboard	"It stores a lot of stuff... if I could have a phone like this, I wouldn’t be walking with an exercise book because whenever I go to the hospital, I must write the date and tick by myself” (KIDH).

a mHealth: mobile health.

b KIDH: Kibong’oto Infectious Disease Hospital.

c KCMC: Kilimanjaro Christian Medical Centre.

Recognized benefits included enhanced convenience, reduced need for transportation and associated costs, and opportunities to receive additional, timely support from peers and providers. Patients generally described the features as intuitive and simple to understand, though some felt other patients may struggle depending on their literacy level. Patients noted that even for those without smartphone access, most would be eager to participate in the program if offered, with adequate upfront training “to the public” (eg, group seminars or campaigns), and one-on-one training suggested over a period as long as a month to ensure understanding.

Several concerns were identified related to feasibility and personal costs to the patient, including limitations in consistent personal access to smartphones and continuous data and internet connectivity:

R: “*Hmm, I really don’t know because even if you install it in my phone, when I’m out of bundle if you send something I will not be able to see it… other things might come for the other days maybe I will find them after a week when I buy bundle, how will that work?”*

[Patient, KIDH]

Among existing platform features, patient-provider communication was most frequently coded, with many patients noting the benefit of communicating with care team members in between visits, addressing concerns as they arise (eg, side effects), and receiving support. One patient noted that this type of “virtual clinic” has already been commercialized by individual doctors within the context:

*…I saw in the social network (WhatsApp), there is one person who has opened something like a clinic, he used to provide advice…and he used to tell them to pay around fifty thousand (Tsh)…*

[Patient, KCMC]

Perceptions of the peer message board feature were the next most frequently coded. Patients noted the benefits of a private space to give and receive support when the illness can be so isolating, and opportunities for shared learning with peers sharing their own experiences with treatment challenges. Patients note providers should also have access to the chat, which was the case for app implementation in Siberia but not in the United States [9]. Appointment and medication reminders and daily “check-ins” were found to be desirable features to support adherence, but it may be overwhelming to receive frequent information through the platform.

Patients noted already using internet resources (eg, Google [Google LLC]) for health information, and that tailored educational resources within the app would be desirable. Patients found the laboratory dashboard feature superior to current methods to track their own laboratory results (eg, “exercise book”). Patients provided several suggestions for additional novel functionality, including expanded self-monitoring options (weight, vitals, symptoms, and laboratory results beyond tuberculosis-specific results) and a feature to send photos to providers along with messages. They also suggested functionality for convenient referral of peers and contacts with concerning symptoms for tuberculosis screening and treatment, and asserted the importance of the availability of app features in multiple languages.

### Determinants of Tuberculosis Care: Providers and Staff

Determinants of care engagement and medication adherence that emerged during provider and staff interviews are summarized in Table 5.

**Table 5.** Determinants of tuberculosis care, experiences with current care delivery, and medical needs: provider/staff interviews. Themes are listed in descending order with subthemes and supportive quotations, based on the number of participant interviews coded with the theme (I=interviewer, R=respondent).

Theme	Illustrative quotes
Determinants of tuberculosis care	
Patient knowledge	"Others just fail to adhere to it on time because they ignore, there are those people when they see that there is some improvement in the signs and symptoms, they feel as if I have probably recovered, do you see? So he ignores to take medicine” (quality officer, KIDH^a^).
Reminders (medications, apps)	“There is a way to go to chew the bister pack aah, look at the date that he has taken the medications and the medications that have been used so, you will see there if you see he should have taken even 20 tablets but you go you find there are 12 which means there is a poor adherence in some days either he did not take it at all or he took some doses. But other digital methods are like reminders, there are devices that are given that give an alarm to remind them” (laboratory scientist, KCMC^b^).
Stigma related to tuberculosis	
Family members	“Some might find themselves in families where others don’t have that illness, so when it’s discovered that they have the illness, they are stigmatized. Therefore, they might take their medication and go somewhere secluded to take it, so they won’t be seen. Sometimes, they skip medication altogether” (laboratory scientist, KIDH).
Community, societal perceptions	“Then after a while I talked to that woman just like half of an hour and she understood me and said that, ‘I was thinking that he acquired by doing prostitution the time he was doing business,’ then I told her that TB is transmitted through breathing“ (community liaison, KCMC).
Cost of care, incentives	“Now, you find that a patient fails to afford and his health is weak, meaning that if he was someone whose health was the source of his income, he goes to do physical works and get an income so, you find that those works are going slowly and there are treatments costs to pay so, you find that in he comes to some appointments but the tests are not done” (laboratory scientist, KCMC).
Patient self-efficacy, motivation	I: “Do you think if you set an alarm to a patient so that he/she can remember his or her time for taking medication will it help in adhering to treatments?” R: “Aah, not much if we do not deal with the mindset of the patient because when you look for example even when you look, we always have that important thing that you are supposed to do and you have set an alarm for getting up but still you felt tired so, the main thing is to deal with the mindset of a person” (laboratory scientist, KCMC).
Patient-provider relationship	“I think it will help especially for some patients though there is always a minor difference because we tried to look on the difference between a patient who is reminded and the one who is not reminded and found out that the difference is very low due to the trust in the health care provider” (physician, KIDH).
Transportation access, distance to clinic	“Yes, we need a system in place for tracking the patients which will be able to link the patients and the health care workers it will be very supportive in management and adherence also it will be easier for these health center to track the medications reports rather than asking the patients to attend every time and also it will serve on the issues of transport cost” (laboratory scientist, KCMC).
Digital adherence tools	"There are systems…when a patient opens a box you get signals that the patient has taken the medication though it doesn’t give you 100% assurance that the patient has taken the medication. Because he can just open the box only or if you have sent him a message he can receive the message but will he really take his medication so still it doesn’t give you 100% assurance. And in those other systems the only method that we were using to know if the patient has taken his medication is when we see the patient after 2 weeks or 2 months and test the blood so we see drug concentration in his blood, there is where we find out whether he was taking medications or not” (physician, KIDH).
Tolerance to medications, side effects	"Therefore, the medications become stronger than their bodies so they get those side effects whereby you may find that the patient is not telling you so if you go and investigate you will discover that he decided to stop taking them or to skip some days because he wants to help himself” (physician, KIDH).
Frequency of medical visits	“Our country is a poor country and most of the people depend on working here and there to get daily bread and these too many visits of course they waste time for taking care of families which depend on us...when these visits become too much, they make people not attend as required” (laboratory scientist, KCMC).
Experiences with current care delivery and medical needs	
Monitoring of patient adherence over treatment course	"Yeah, the challenge of DOT, we have not put in the system that the doctor provides medication to the patient, the patient is the one who stays with the medication. The main role of a doctor is to ask the patient ‘did you take the medication today?’ So, not all the time that the doctor will be able to see the patient” (physician, KIDH).
Laboratory diagnostics for tuberculosis	“Ok, basing on my experience there is not a big challenge during the time of taking sample, samples are taken well and tested…but in releasing the patient result is where the challenges comes…You can find out that the patients does not make follow up of their result, when it occurs they come too late and others they don’t make follow up at all, so sometimes it leads into delay in treatment” (laboratory scientist, KCMC).
HIV and tuberculosis care integration	"Okay aah, now there is one challenge for TB patient, to come to the clinic since these places have been joined for TB and HIV patients so, you find that they feel that when they go there, people will know that they have HIV. So, you find that they get hard time so, we encourage them that ‘Don’t mind what people think about you, mind about your health’" (laboratory scientist, KCMC).
Off-hours calls	I: “Maybe they don’t know if you’re not in the work that day?” R: “They use to call; you know what ...we Tanzanians have that kind of love you can’t say that…!” I: “That I’m not present today.” R: “For he has called you means that the person needs help.” I: “Yah, and maybe your phone was the one to save his life.” R: “So, most of the time we don’t set a limitation to them, we just tell them that when you see something unusual you can call or you can come even if it’s not a clinic day” (nurse, KCMC).
Community health, outreach services	“At my telephone I have more than fifty patients for sure…I use to communicate with them so, as to know how they are doing” (community liaison, KCMC).
Monitoring of medication tolerance, side effects over treatment course	“I tend to have their contact details, so he will communicate with me if is experiencing any challenge even those side effect and I can tell him that…’you have to improve the food you’re taking’” (community liaison, KCMC).

a KIDH: Kibong’oto Infectious Disease Hospital.

b KCMC: Kilimanjaro Christian Medical Centre.

Patient knowledge was described as critical for encouraging long-term adherence and preventing early treatment discontinuation, including around other facets of health (eg, maintaining adequate nutrition or cessation of smoking), with variable literacy levels observed among their patients. As with interviews with people with tuberculosis, daily medication adherence and the patient-provider relationship were highlighted, noting both were insufficient without adequate patient motivation and self-efficacy. Patient-provider relationships were described as patient-centered with shared decision-making by some, and more directive approaches by others.

Stigma was widely described as a barrier, influencing the degree of social support received by family members and community members. Providers described stigma fueled by inadequate understanding of how tuberculosis is acquired (eg, misperceptions around sexual transmission or assumption that a patient with tuberculosis automatically has HIV) and further transmitted (eg, even after the patient has been on treatment for some time). Several providers stressed the importance of proactive counseling and education of the patient and family members to reduce stigma, foster family support, and reduce early treatment discontinuation and patient isolation. Cost of care (eg, transportation fees or laboratory tests) was a major determinant, as with patient interviews. Providers described competing costs (eg, feeding their families) and the difficulty of frequent in-person medical appointments for maintaining employment. Several providers mentioned that incentives (transportation or food vouchers) would greatly improve care engagement.

### Care Experiences and Unmet Medical Needs: Providers and Staff

A variety of methods were described to encourage medication adherence and appointment attendance (Table 5). Provider-initiated phone calls are the primary method. Providers and staff note that patients can be difficult to reach (data bundle runs out or family members must be contacted), and repeat outreach attempts are time-consuming for providers. Other methods of adherence monitoring were described with some concerns about efficacy, including blister packs (patients bring in to visits inconsistently) and digital adherence tools (eg, patient opens an electronic pill box but does not take the medication). Providers and staff noted limitations with home-based DOT, or a trade-off between patient access and accurate monitoring of daily adherence.

Experiences with implementation of laboratory diagnostic testing were highlighted by many providers, including challenges with releasing results to patients, causing delays in treatment initiation, and additional service delivery costs when patients cannot be reached by phone or do not return to the facility to receive results (eg, staff must arrange transportation to find the patient out in the community). Respondents at both sites noted care integration for HIV and tuberculosis facilitates appropriate medical management (eg, adjusting HIV medications for drug-drug interactions), but that collocating care has resulted in additional HIV-related stigma for people with tuberculosis mono-infection. Providers and staff at both KIDH and KCMC described off-hours calls, noting they are open to receiving calls outside of work hours or seeing patients who present outside of the providers’ scheduled clinic hours, as it contributes to better care, but it presents a burden on their time and energy. Providers and staff described current outreach services performed by community liaisons, including moderating patient support groups and education seminars, checking in with phone calls, doing home visits, and contact tracing.

### Smartphone and Internet Access, Ownership, and Familiarity: Providers and Staff

Personal access to a smartphone was reported by all 11 providers, with broad access among peer and family networks. As with patients, they described most frequently accessing the internet via data bundles over Wi-Fi, both for work and personal use. High levels of comfort and familiarity with smartphone apps were noted, including for personal use (communication with peers, social media, and news) and for work (reviewing medical literature, clinical guidelines, communicating with patients by phone call, and with colleagues and coworkers by WhatsApp or calls). Providers and staff noted use of their personal smartphone over other devices (desktop computer or laptops) for these work tasks, and the inability to access hospitals’ digital systems via these devices. The cost of bundles is also often not subsidized (with spending reported between 10‐100,000 Tsh or ≈3.8‐38 US $/mo).

### Patients’ Implementation Determinants: Provider and Staff Perspectives

Determinants of implementation emerging from provider interviews are categorized for salient domains, subdomains, and constructs using CFIR version 2.0 [28] with illustrative quotations (Table 6).

**Table 6.** Implementation determinants categorized using CFIR^a^. Additional themes categorized using CFIR 2.0. Themes and subthemes are shown with illustrative quotations for the most frequently coded domains and constructs (I=interviewer, R=respondent).

Theme (CFIR domain/construct)	Illustrative quotes
Innovation	
Self-monitoring check-ins	“Because I’m sure that if I will always sign his mood swing at the end of the month it can be like self evaluation. So, all month it’s all twenty days I was very angry, I had stress, maybe something is not ok with me. Maybe he can make a self evaluation on his life style” (nurse, KCMC^b^).
Patient-provider communication	“Therefore, I think that something can be very helpful because when the patient has a challenge, it will make it easier for him to be able to communicate with the service provider in the hospital, so that he can present what he feels and if that thing will not be a big thing, it means that the patient can get his results in time and he will be advised to maybe do one, two or three, life went on and he just continued with his medicines” (quality officer, KIDH^c^).
Reminders (appointment, medication)	“On my side I will be able to catch up with the patients right there because he will have already acquired that communication knowledge. And it will be very good if it will be directing him about the time of using medicine, since it will make it easier for me that there will be no need to make a lot of follow up” (nurse, KCMC).
Peer messaging board	“Yeah, I think even on the side of mindset it will help in building people because sometimes you can feel like you are alone but if you can communicate with each other without recognizing your identities like that and communicate something, it may help, I think it is so important” (laboratory scientist, KCMC).
Laboratory results dashboard	“After the patient has got the result there is the need of giving him counseling psychologically…Sometimes they refuse to take (the medication) but if you take time to talk with him with a kind language, the patient will accept to take the medicine and will see that you care about him” (community liaison, KCMC).
Educational resources	I: “Do you think that if we put information queries, resources information…do you think it is useful?” R: “It is to give detailed instructions on drug education, correct use, what to use, what not to use, how long not to take, with empty stomach or with food. There is a lot of information about medicine” (hospital attendant, KIDH).
Inner setting	
Compatibility	
Clinical processes, workflows	I: “Are there any changes we will be required to make?” R: “I don’t think if there is the need because many health system nowadays are using these information system so, I think what is required is to have the means of linking the information…and it should be between the app and all the systems being used at the hospital like the patient registration system or the one which provide the result from the lab, so when the results are out the patient can receive it through the app” (laboratory scientist, KCMC).
Provider workload	“If a group (of patients) can have more than one doctor so for the one who will be disturbed much due to a problem, it means that when a patient will be raising any issue, the one who is free at the moment will have the ability to respond and help the patient while the other one is busy with the work” (physician, KIDH).
Patient-provider relationships	“There are those who prefer to make decisions through shared- decision making and others are more directive” (community liaison, KCMC).
Available resources	
Internet access in clinics	“The one that affects much is the system, currently we see our government has removed paper works and it is planning to use the internet therefore, moving from one place to another becomes a challenge. And when we look currently, the electricity issue you may find that someone is at the reception while waiting for the receipt then suddenly the power goes off and when it comes back the system has to restart so, you find that the patient is getting late directly and when it comes to the laboratory side it’s the same thing” (nurse, KIDH).
Provider access to smartphone, data for work	R: “Most of the time I always buy for myself but sometimes due to our works the project may provide bundle for the staff. Yeah, sometimes the cost of bundle becomes higher than earning because I can even use 100,000Tsh. Per month.” I: “And what forces you to buy bundle, is it because of works or because you want to get social services? R: “Mostly it is work” (physician, KIDH).
Other devices (laptops, desktops)	"And when the system was introduced, did they come up with all the resources like the new computer, everything? R: No, you find someone with a laptop is connected to the system on their laptop but there are those desktops for example, in the registration unit, they have desktops that have the system, if you come to the laboratory, they also have them, the wards have them too but if it’s an individual you also have the ability to have them even if it is a doctor, he/she has the ability to connect” (nurse, KIDH).
Existing electronic record/digital system	I: “Have you ever used your phone to enter into the system?” R: “No, for us you cannot use a phone to log in to the system.” I: “Is it laptop only?” R: “Yes, it is a laptop for a system that is made by the hospital, there is a password and user name that you have to login with.” I: “And if you are outside the center, can you log in to the system?” R: “No” (nurse, KIDH).
Individuals: characteristics	
Innovation deliverers	
Motivation: provider buy-in	“On my side I will have my own goals which I will set and when I’m implementing I will ensure that I meet the goals. That’s why I told you that I will make sure if there are patients who want to stop using medicine I ensure that I persuade them until they accept to use…So, we are required to have clear communication, you know sometimes if you have challenges you have to give it out because without doing that you will not reach the goals” (community liaison, KCMC).
Capability: knowledge, skills, self-efficacy	“Most people use smart phones and there are people who use things which might be a complicated application, more than this which we saw and most of them are youths who are good in these network issues so, I don’t think there is an issue which may hinder them from operating it” (laboratory scientist, KCMC).
Need	“We see it as something which is going to increase ability to reach many patients, it also reduces wastage of time because caregiver could not reach every one because they were doing home visit but this one helps because there are things which are completed through application, and also it reduces the number of staff who were supposed to visit patients” (laboratory scientist, KCMC).
Opportunity: availability, scope, power	I: “Mmmh charting this of course you said this is not much but you feel doctors, are they eager to receive (a) message at night maybe.” R: “Mmmh for the doctor I don’t think so.” I: “So, what is the best way to make sure that this charting function works?” R: “I just think they should be given to nurses…nurses have something that… it’s patience” (quality officer, KIDH).
Innovation recipients	
Capability: knowledge, skills, self-efficacy	“I will be more curious to see the acceptability of the application between the people who had different education level, also basing on the areas for example those who are from the town areas are the one who are accepting it more or are those from the rural areas, and also I will be curious to see those who does not accept the app or they don’t want to use it what is their views” (laboratory scientist, KCMC).
Motivation: patient buy-in, commitment	“There will be challenges which you will face, but the thing which is required is the provision of education to the patient because when you look on HIV there other places they consider it as the shameful disease so, there will the challenges on the side of patients and the first question they will be asking; how safe my information will be? So there will be resistance like those but if you out the good education they will accept it well” (laboratory scientist, KCMC).
Opportunity: availability, scope, access, power	R: “Here, for those I’ve been serving during this time, very few come with basic phones, maybe a student who won’t bring a smartphone at all. Or maybe an elderly person who might have a very small phone.” I: “So, it’s this middle generation.” R: “Almost all of them come with smartphones” (laboratory scientist, KCMC).
Access to internet, data, smartphone	“On the issue of network maybe for those who are from the rural areas, and there is the issue of the patient’s ability of having smartphone because I believe that the app will require the use of a smartphone, right?” (laboratory scientist, KCMC).
Financial means	R: “There are those patients who are capable to afford the cost because being patient doesn’t mean that they don’t have a source of money.” I: “And you find it mostly in which age?” R: “You find those who are being employed in age around thirty up to thirty-five. But there are those who can’t afford in which maybe when there is a study like this they can be supported with a small amount for bundle after assessment” (nurse, KCMC).
Individuals: roles	
High-level leaders	R: “He is a chief, he has developed different systems in providing education on TB, HIV and so on, so he has been coordinating.” I: “What is his profession?” R: “He is a doctor but he has been assigned in the training department. Therefore, the training department is the one that can help you succeed” (physician, KIDH).
Implementation leads	“There are those leaders or doctors even the whole management at the clinic like matrons should have this all information and the time of providing the seminar which we will have to be trained there is the need of having maybe one doctor to represent all other, there should be nurses to represent other nurses, there should be the health care workers and even few patients” (community liaison, KCMC).
Implementation team members	“You know because the ICT are more technical they will be responsible too but when you want to look on patients I think the service provider is the one who are capable of convincing the patients on how to use the app and it’s advantages because I think it’s very hard for IT personnel to explain to the patient about the adherence” (laboratory scientist, KCMC).
Implementation facilitators	“The technical team because they’re one who knows the system…So, they’re the one who come to teach us. I: And things like seminars who are responsible to teach you? R: Technical team is the one who is responsible” (nurse, KCMC).
Implementation process	
Planning	
Training: innovation deliverers	R: “No, I can say that at a certain rate because in the system too there are regular changes, when it happens that there is an update you find that we are given instruction in general but if for example, there is a session maybe all the staff are needed then it happens that maybe one person is missing and it happen that you are already trained you have to train someone so, sharing information from someone who is trained to the other who is not trained will lead to improper understanding” (nurse, KIDH).
Training: innovation recipients	R: “Perhaps through seminars, advising the patient, that this app or this phone I’m giving you, will give you contact with you and the doctor…usage, I mean how to use this phone, it’s you and the patient and you’ll get your information through the doctor” (laboratory scientist, KIDH).
Management: planning	“I think it is information, because as you know the one who are responsible mostly making decisions are management so, if the management would have the right information they can be able to notice the advantages from the system so, if the right information will be distributed to the responsible team I think what will be next implementation only” (laboratory scientist, KCMC).
Engaging	
Leadership	“The first thing is for the high rank leader to accept the system, then he/she will know the subordinate to deal with that system. The leader has to accept the system followed by the IT. But in understanding the system is for the IT but in accepting the system is the leader” (nurse, KIDH).
Clinical providers, staff	“You know because the iT are more technical they will be responsible too but when you want to look on patients I think the service provider is the one who are capable of convincing the patients on how to use the App and it’s advantages because I think it’s very hard for IT personnel to explain to the patient about the adherence” (laboratory scientist, KCMC).
Other departments	“Health care workers for supervising are present too…The IT personnel, when he/she understands since he/she is always around the hospital even when somebody faces a challenge it will be easy to consult him/her” (nurse, KIDH).
Reflecting and evaluating	”It will depend on you have being prepared example is when I’m there at OPD (outpatient department) with patients it will be better if you will be attending there even twice per week, by doing that it will help because you will be in position of seeing the challenges direct since it’s the new thing we are try to introduce. And we will be able to solve those challenges on time…Yes you must be there for monitoring and for checking if the program is going as it was planned. And when you see it going contrary you correct it, so as we can know where we are required to make improvements” (community liaison, KCMC).

a CFIR: Consolidated Framework for Implementation Research.

b KCMC: Kilimanjaro Christian Medical Centre.

c KIDH: Kibong’oto Infectious Disease Hospital.

Perspectives on various platform features are categorized within the “innovation” domain. Positive perceptions of acceptability surrounding the design of the app’s features were coded among all 11 provider and staff interviews. Providers and staff noted potential benefits including minimizing adherence challenges and treatment interruptions, reducing the frequency of in-person visits and patient travel, and the potential to enhance existing community outreach efforts. Positive perceptions of usability or user-friendliness were coded among 5 interviews based on the platform overview provided.

Providers and staff describe the utility of daily check-ins of mood, stress, and medication adherence*,* which should be effectively summarized for all members of the care team. They emphasized the importance of mood and stress on medication adherence and current limitations in assessing with intermittent face-to-face visits. Some respondents note that reminders should not be overly frequent, but can, over time, increase motivation, as well as create an opportunity for providers to initiate discussions around observed increased levels of stress. Suggested additions included a video feature for observed doses to ensure adherence beyond self-report (eg, video-DOT), check-ins for symptoms and side effects, and additional reminders to eat nutritious meals to support medication tolerance.

Participants noted the benefits of direct communication between people with tuberculosis and providers and staff through the in-app messaging tool, including a “pool” of providers that can respond if the primary provider is unavailable. Providers and staff participants, such as people with tuberculosis interviewed, noted that the peer messaging board creates an opportunity for shared learning and building community. Educational resources were described as useful to demonstrate the benefits of adherence and set expectations around possible side effects. The laboratory results dashboard was felt to be desirable to reduce delays in treatment initiation/modification and prevent loss to follow-up following an initial diagnosis. One respondent noted the app could improve upon the current lack of security for current laboratory entry, whereby multiple people can enter a laboratory result without tracking who modified it and when. Direct delivery of a positive result/diagnosis via the app without associated counseling was a shared concern among respondents, noting potential implications of direct delivery of laboratory results to people with tuberculosis via the dashboard without appropriate counseling.

Relevant themes emerged for the “inner setting” domain—the hospital/clinic settings where providers/staff are employed—including compatibility with clinic processes and available resources (eg, smartphone, electronic device, internet access at work, or as described previously). Instability of electricity and power outages were noted as an issue that might impact implementation. Providers and staff noted that the program would need to be well integrated early on between the clinic, pharmacy, and laboratory settings. For the “individuals” domain (individual characteristics and roles), providers and staff (innovation deliverers) generally noted feeling confident in their own ability and those of their colleagues to effectively use the app, possessing appropriate knowledge and skills. Several respondents noted that, in addition to meeting care delivery needs, the app may allow for increased capacity to reach patients, increased efficiency, and time saved on other outreach activities.

Provider and staff availability to engage with the app was felt by some to be contingent on additional funding and engagement of multiple collaborating team members. While some respondents felt physicians should lead program implementation and field concerns from patients via the app (eg, direct messaging or chat board moderation), others felt nurses or community health workers might be better suited based on their current roles. The IT, training, and quality departments were noted as key for implementation facilitation and support, and that patients should be represented in decision-making processes. A physician provider noted that additional compensation should be considered to incentivize involvement. Providers and staff anticipated patients (innovation recipients) would be supportive and capable of receiving the intervention, contingent on factors such as age, place of residence (eg, rural vs urban), educational level, and financial means, each shaping personal smartphone and data bundle access and “digital literacy.” Some anticipated challenges include personal cost to the patient (eg, bundle to support usage or need for provision of smartphone), privacy concerns (eg, by whom and how will the patient’s data be accessed), and a need for translation to multiple languages (eg, English, Kiswahili, or local languages).

For the “implementation process” domain, planning and continuous engagement of high-level leadership and IT staff, in addition to patient-facing staff, were highlighted by providers, as well as proactive training and engagement of patients to ensure adequate awareness and buy-in. The importance of a physical presence (eg, direct observation) in implementation settings early on to assess progress and challenges in real-time was also noted by one provider.

## Discussion

### Principal Results

Salient themes emerging from our contextual assessment are summarized in Figure 2. Stakeholders engaged by this study demonstrated a high level of perceived acceptance for the proposed app and program as described, with significant barriers emerging related to feasibility in this “real-world” context. Smartphone ownership was uniformly described among providers and staff members and their networks, whereas people with tuberculosis described considerably lower access. Our findings suggest that a provider-facing smartphone tool to assist with interstaff communication, sharing of results, and other functionalities independent of hospitals’ electronic systems was perceived to be feasible and acceptable. Current bidirectional features requiring smartphone and data bundle access may also perform well in funded pilot trial conditions for a select patient persona (urban residence, young to middle-aged, etc); however, broad real-world reach and sustainability of patient-facing smartphone tools would require significant infrastructure development and financial support within the context.

Our application of CFIR in this study was limited to the providers and staff members at KCMC and KIDH, without representation of high-level leadership or external stakeholders (eg, government, network providers, etc) who could elaborate on potential mechanisms to support sustainability and financial viability in the context. There is growing evidence for cash transfers and other forms of financial support to provide person-centered tuberculosis care, among which digital access could be further prioritized (eg, provision of a smartphone and data bundle for the duration of treatment) [38,39]. The phenomenon of widening health care inequities through the broad roll-out of person-centered digital health interventions—without a parallel process of design and planning to support implementation and reach among individuals with lower digital access—is increasingly observed as broader population-level trends suggest growing digital access [40,41]. Our findings show that barriers to tuberculosis care intersect with digital access barriers, stressing the importance of conducting thorough contextual assessments to inform equitable implementation, as opposed to relying on broader-level population trends.

PositiveLinks features were designed based on the IMB skills model. The IMB model posits that to change a habit or promote health, a person must use cognition to understand information and build motivation for action and deploy behavioral skills to reduce an unhealthy behavior. PositiveLinks provides information about daily medication adherence and helps the user track behavior over time, enhances motivation through secure messaging with providers and social support from peers, and encourages behavioral skills in HIV care self-management via self-monitoring of mood and stress in addition to medication adherence. Interacting contextual determinants identified in this study are highlighted as both (1) impacting individual abilities to understand information and build motivation and (2) integral to the SAT that further guides PositiveLinks platform design, by shaping the contextual influences and social interactions that further influence individual behavior change as described by the IMB model (Figure 2).

Existing theory-based platform features developed for PositiveLinks address many needs endorsed by people with tuberculosis and providers in this context (eg, low barrier communication, direct in-app laboratory result release, peer support, educational resources for stigma reduction, and prevention of early treatment discontinuation). Several additional suggestions for novel features and adaptations were provided for patient-facing features specific to tuberculosis care in the context, such as video-DOT, which has some evidence for efficacy over traditional DOT, but is limited by access and connectivity barriers [3]. Added self-management features (eg, check-ins for symptoms, side effects, or reminders for nutrition intake) and availability in multiple languages were suggested by both patients and providers and will be considered in future prototypes. To date, most platforms tested or disseminated in Sub-Saharan Africa focus on daily medication adherence support and monitoring. This study indicates that more intensive digital wraparound support is desired and could enhance the impact of digital adherence technologies for tuberculosis care, mitigating barriers related to additional mechanisms of behavior change related to patient knowledge, motivation, activation, and related behavioral skills of self-management and peer support.

Several adaptations for the associated implementation strategy were identified for consideration for future implementation of PositiveLinks and other mHealth technologies to support tuberculosis treatment among people with tuberculosis in this context (Figure 2), distinct from prior implementations in other contexts [9]. Existing usage and degree of digitization of records was described in multiple themes across interviews (eg, use of personal print records for visits, including “exercise books,” digital records accessible only by devices located within clinics) and represents an important consideration for digital health intervention compatibility within care settings in the context. Additional distinct emergent needs identified for the context will need to be considered for intervention and program adaptation and implementation among innovation recipients (digital literacy, access, and income) and deliverers (integration between departments, personal provider and staff cost of device usage, existing provider and staff burden, and community outreach infrastructure).

### Comparison With Prior Work

Our findings related to broader determinants of tuberculosis care, care experiences, and unmet needs in the context align with those previously reported within Tanzania and similar contexts to date, including barriers related to stigma and social support, transportation, geographic access to facilities, indirect costs (transportation, nutritious meals, and laboratory results), and competing demands of employment and basic needs [42-46]. Our study expands on limited literature by exploring real-world smartphone ownership, access, and familiarity within the context and perceptions surrounding the implementation of smartphone-based and other mHealth technologies to support the delivery of person-centered tuberculosis care in the region [47,48], as well as key considerations for designing compatible implementation strategies for the context.

### Limitations

Our study had several limitations. The sample size for each stakeholder group is the result of a combination of thematic saturation reached early on in recruitment and limitations surrounding study funding and staffing, with a long recruitment time needed—a common barrier for rigorous implementation research [49]. Certain provider and staff roles were consequently underrepresented (eg, physician) or overrepresented (eg, laboratory scientist). We included people with tuberculosis up to 5 years posttreatment to be inclusive of potential long-term care experiences and consequences of incomplete or poor-quality tuberculosis treatment that take time to emerge. Accounts for participants farther out from treatment complications may be limited by recall bias, which could limit accuracy and comparability with more recently treated participants. We also elected to apply CFIR only to provider and staff interviews, as we felt that other probes related to context adequately captured salient CFIR constructs and domains for people with tuberculosis (eg, patient needs or characteristics). Future studies to develop prototypes and further engage stakeholders in program design and implementation should deepen the involvement of both people with lived experience and stakeholders external to potential implementing organizations.

### Conclusions

A contextual assessment conducted among stakeholders at KIDH and KCMC in the Kilimanjaro region of Tanzania demonstrated high acceptability for the proposed adaptation of the PositiveLinks mHealth, smartphone-based strategy to deliver multidimensional, person-centered care of tuberculosis within the context. Several assets were identified to support PositiveLinks implementation, including a robust existing community outreach infrastructure and provider and staff commitment to patient care outside of scheduled appointments. Providers and staff demonstrated high subjective capability, motivation, and need, though several shared inconsistent current infrastructure to support smartphone app use for clinical care (power outages, network availability, and unsupported cost of personal device usage). Implementation determinants challenging the feasibility for patient-facing tools (baseline digital access, literacy, and associated costs among people with tuberculosis) necessitate further adaptations to the existing PositiveLinks core implementation strategy components.

## Abbreviations

CFIR: Consolidated Framework for Implementation Research
DOT: directly observed therapy
IMB: information-motivation-behavior
KCMC: Kilimanjaro Christian Medical Centre
KCRI: KCRI
KIDH: Kibong’oto Infectious Disease Hospital
mHealth: mobile health
SAT: social action theory
